# Effect of Addition of Chokeberry Juice Concentrate and Foaming Agent on the Physical Properties of Agar Gel

**DOI:** 10.3390/gels7030137

**Published:** 2021-09-10

**Authors:** Ewa Jakubczyk, Anna Kamińska-Dwórznicka

**Affiliations:** Department of Food Engineering and Process Management, Institute of Food Sciences, Warsaw University of Life Sciences (WULS_SGGW), 02-776 Warsaw, Poland; anna_kaminska1@sggw.edu.pl

**Keywords:** foamed gel, texture, structure, colour, acoustic properties

## Abstract

This study aimed to determine the effect of the addition of chokeberry juice concentrate (CJC) and foaming agent (egg albumin) with different percentages on the selected physical properties of agar gel. The agar gels with the addition of 5, 10, and 20% concentrations of chokeberry juice concentrate and with fructose addition were prepared. In addition, the foamed gels with different concentrations of egg albumin (in the range 0.5–2.0%) and CJC were produced. The water content, colour, density, hold-up and some mechanical and TPA (Texture Profile Analysis) descriptors as well some structural and acoustic emission parameters of non-aerated and foamed gels were analysed. The addition of CJC changed the colour of agar gel with fructose, the attractive appearance of the aerated gel was also linked with the addition of concentrate. The addition of 20% of CJC and foaming agent created samples with very low hardness, cohesiveness, and gumminess, and the structure of the aerated samples was characterised by the larger bubble diameter and the wider distribution of their size. The more promising texture and structure properties were obtained for samples with aerated gels with 5 and 10% addition of chokeberry juice concentrate.

## 1. Introduction

Gels play a key role in the creation of many food products such as jelly desserts confections, tofu, or dairy products [1]. Confectionary gelled products can contain different sugars such as sucrose, fructose, glucose, inverted sugar, or glucose syrups. The addition of sugars changes the taste of gels and affects the decrease of water activity and stability of the final product [2]. Among the common hydrocolloids used in developing fabricated and confectionary food gels, agar-agar can be an effective gelling agent which can form a thermo-reversible and stable gel over a wide range of temperatures [3,4]. Additionally, compared to other gelling agents such as gelatine; agar is derived from a non-animal source which makes this product suitable for vegetarians [5]. Introducing air or other gases into gel matrix is a method wherein the properties of gels can be modified to affect the structure, texture, colour and mouth-feel of products. Aeration of gels can find application in lowering the caloric density and increasing the satiety of foods [6,7]. Aerated gels have a lower mass than a non-aerated product with the same volume of sample. The reduced density of gels with bubbles may increase a sense of fullness in comparison to a gelled product without a gas phase [8]. 

The incorporation of air bubbles into sol leads to create the porous structure of foam. Food foams can be described as biphasic systems in which a gas bubble phase is dispersed in a continuous liquid phase [9,10]. Foams are inherently unstable and their stability and foaming capacity can be enhanced by the addition of surface-active molecules such as low molecular weight surfactants and high molecular water-soluble biopolymers [9,10,11]. Foams can be made with addition of foaming agents such as Tweens, the mono- and di-glycerides proteins, especially derived from milk and soy [12] as well from egg white and egg albumen [13], but only a few polysaccharides (e.g., modified cellulose derivates and acetylated pectin) are suitable for practical purposes [10,14]. The presence of a foaming agent modifies the rheological properties of interfaces regions and continuous phase which leads to improvement of the structure and texture of foams and their stability [10]. The amount of added foaming agent can also be a key factor affecting the structure of foams. Dachman et al. [15] used potato protein isolate at a concentration of 2.5, 5, and 10% to obtain foamed raspberry puree. It was observed that with increased protein concentration higher values of the overrun were obtained as well as the stability of foam was enhanced. Foams were also prepared from starfruit puree with the addition of methocel 65 HG as a foaming agent. As the concentration of the methocel increased from 0.1 to 0.4% the overrun and the air bubble diameter increased. However, an increase of the foaming agent concentration further caused the opposite effect, as the volume of foam and the size of the bubble decreased [16]. Raharitsifa et al. [10] obtained apple juice foams by the addition of foaming agents (egg white, methylcellulose) at a different concentration. Egg white foams were less stable but showed higher foaming capacity, stronger structures, and smaller mean bubble diameter than methylcellulose foams. Foam stability increased but the bubble size progressively decreased with increasing concentration of foaming agents. 

Novel gelled products can also be obtained by the addition of fruit purees and juices. Tiwari and Bhattacharya [17] prepared gellan gel with mango pulp, which contained a high amount of antioxidant compounds and sugars used as sweetening and texturizing agents affecting the texture of gels. Carrot juice as a source of β-carotene was also added to agar and gellan gels [3]. Some examples of the application of juices and pulps to fabricate gels can be found in the literature. However, studies on the aerated gels and marshmallows with fruit juices and pulps are very limited. Çoban et al. [18] used the barberry fruit extract as a natural colourant in jelly and marshmallow at different concentrations of 1, 5, and 10%. The antioxidant properties of aerated gels were improved by the addition of the extract. 

The incorporation of juices, extracts, and pulps into the gel system may significantly improve the colour of gels and the content of bioactive compounds in a final product. The addition of extracts and juices of dark berries can be very beneficial due to their antibacterial, anti-inflammatory, anti-oxidant, and hypoglycemic properties [19]. Berries contain a wide range of flavonoids (anthocyanins, proanthocyanins, flavanols, catechins) and phenolic acids (hydroxylated derivates of benzoic acid, cinnamic acid) which play a preventive role in the protection from coronary heart disease and cancer [20]. Phenolic extracts from black berries were shown to inhibit oxidation of human low-density lipoprotein and liposome oxidation [20,21]. Chokeberry fruits are a rich source of bioactive compounds and they are known for their antidiabetics properties as well as gastroprotective affects [20,22]. Among the many berries, chokeberry is characterised by the highest total anthocyanin concentration (~1480 mg∙100 g^−1^) and the total antioxidant capacity (~161 μmol of TE∙g^−1^) [23]. Chokeberry fruit *Aronia melanocarpia* (Michx) Elliott belongs to the Rosaceae family and is native to the eastern part of North America. Today, chokeberry is also cultivated in Eastern Europe, and it is used for the production of juices, jams, fruit tea, wine and natural colourants [24,25]. The presence of cyanidine glucosides in the outer skin of chokeberry fruit is responsible for its dark purple colour [20,26]. The fruits are also a source of lipids such as triglycerides and linoleic and linolenic acids [24]. Some studies have shown that the phenolic compounds distribution and antioxidant activity were dependent on a form of fruit product. The pomace had a much higher content of phenolics than juice and chokeberry fruits [20]. In addition, chokeberry capsules and powders contained a considerably higher amount of total phenolics and total anthocyanins than fruit tea and dried berries. The lower concentration of anthocyanins in some chokeberry products can be a result of pH, chemical composition, temperature, or the presence of light and oxygen [25].

The texture-structure properties of food gels are very important, as the functionality of a product is related to its mechanical behaviour during processing and mastication [1,27]. Aerated gels can offer a wide range of advantages including flexibility in texture and structure and many sensory attributes related to consumer acceptance of food products [4]. The texture of hydrocolloid gels can be characterised by several mechanical, structural, and acoustic features perceived by consumers [28]. The mechanical properties of gels can be analysed using different instrumental methods. The large strain deformation method is recommended to obtain mechanical (fracture) parameters that are strongly related to sensory attributes [29]. Some textural properties of foods can also be predicted based on information included in the acoustic signal generated during the deformation of the products. The accelerations evoked in deformed material can be registered by a piezoelectric contact sensor. The acoustic emission method was used to analyse properties of crispy products such as cereal extrudates [30,31], dried gels [32], apples [33] but also sugar foams [34,35]. 

This study aimed to determine the effect of the addition of chokeberry juice concentrate and foaming agent (egg albumin) with different percentages on the selected physical properties including mechanical, acoustic, and structural parameters of agar gel.

## 2. Results and Discussion

### 2.1. Physical Properties of Gels with Chokeberry Juice Concentrate

Gels without the addition of chokeberry juice concentrate were characterised by water activity 0.678. The addition of concentrate in the range of 5–20% caused a decrease in water activity from 0.659 to 0.611 (Table 1). The reduced water activity was related to the addition of CJC. Samples with lower incorporation of juice concentrate contained a higher amount of added pure fructose. It means that the water-binding was different in samples with added fructose and products with the addition of CJC. The composition of fruit concentrates is more complex because apart from fructose they also contain glucose, sucrose, sorbitol, and organic acids [36], in which their presence can also affect the mobility of water molecules in a gel.

The density of gels did not differentiate with the addition of a different amount of juice concentrate (Table 1). The dry matter of the sample was similar, the gels contained 56.5% dry ingredients (added fructose, agar powder, solid ingredients of concentrate), thus the density of prepared gels was similar. In other research, the density of gels with the addition of a lower amount of agar powder (0.86% *w/w*) and fructose (55.5%) was about 1290 kg∙m^−3^ [37].

The colour of gels was related to the addition of CJC (Table 1). The gel without of CJC was very bright (L* = 96.0) since the parameter L* of 100 represents white. The incorporation of concentrate caused a decrease of the lightness L* variable from 24.1 to 4.1, which can be related to the addition of a higher amount of deep colour pigments typical for chokeberry such as polyphenol compounds included anthocyanins. Tolić et al. [25] observed the low values of L* from 0.52 to 15.0 for chokeberry juice (produced by different manufacturers), which indicated that samples were very dark. Similar vales of the L* of chokeberry was noted by Ochmian et al. [38]. Authors also measured the lightness of chokeberry pulp and the L* values ranged from 24.9 to 35.8 [38]. The intensity of colour was dependent on *a and b* values. The +a* value (redness) was the highest for gels with the 20% addition of concentrate. Positive a* values were also observed in chokeberry juices [25], fruits [38] and in chokeberry powders [39]. The decrease of CJC addition caused a slight decrease of a* values. The b* value provided the information of position of the colour gamut between yellow (+b*) and blue (−b*) [38]. The higher addition of juice concentrate in gels led to a decrease of b* values from +1.1 to −0.6 which can be related to the higher amount of blue-colouring substances in gels with 20% concentration of CJC. 

The mechanical deformation tests of gels showed that the addition of CJC caused a decrease of fracture force and strain as well as Young’s modulus (Table 2). Additionally, the TPA parameters such as cohesiveness and gumminess also decreased with the presence of the concentrate. Cohesiveness indicates toughness during eating and the degree of difficulty in destroying the sample’s structure. Cohesiveness describes how well the product withstands a second deformation relative to its resistance under the first deformation during TPA tests. Gumminess reflects the energy required to disintegrate a semi-solid food to a state at which it can be swallowed [40]. The results obtained by Banerjee and Bhattacharya [3] showed that the fracture force was lower than 10 N for gellan-sugars gels and the incorporation of carrot juice increased the hardness of gelled system. Also, none of these gels did behave as brittle samples as the fracture strain was higher than 30%, which is consistent with the present study. The texture of agar gels with the addition of carrot juice was different than obtained for gellan gels because the presence of the juice caused the decrease of fracture force [3] which is similar to the results obtained for gels with the addition of CJC. The fracture force and strain of gellan gels with the addition of mango pulp decreased with a higher amount of added pulp. The addition of mango pulp rendered the gel structure weak due to interfering in the formation of junction zones. The sensory cohesiveness of mango gels also decreased with a higher amount of added pulp [17]. The chokeberry gels also showed lower values of cohesiveness and gumminess with an increase of concentrate addition. The presence of a high concentration of mango pulp caused the delay in gellan gel formation process which affected the reduced strength of gel [17].

The acoustic properties of gels with CJC showed that total acoustic energy and number of AE events significantly decreased with the higher amount of added concentrate (Table 3). The obtained gels showed a higher failure strain which means that a highly deformable network was created. The structure of gels affected their acoustic behaviour during deformation The samples were less brittle which generated a lower number of cracks with lower acoustic energy.

### 2.2. Selected Physical Properties of Gels with the Addition of Albumin and Chokeberry Juice Concentrate

The water activity of aerated gels with the addition of CJC from 5 to 20% ranged from 0.651 to 0.607 (Table 4). The water activity of samples with different concentrations of albumin (Table 4) and also without albumin (Table 1) at the same amount of added concentrate did not differ. Tan and Lim [41] observed water activity in the range 0.590–0.646 for marshmallows with different types of gelatine. Water activity for most confections falls below the critical values for microbial growth. The water activity of marshmallows is around 60–70% but it depends on the type of marshmallow products and processing conditions [42]. 

The concentration of albumin affected the gas hold-up values of gels (Table 4). The increase of concentration of foaming agents caused a decrease in gels density and an increase of hold-up values. It means that more of the gas phase was incorporated in a gelled system with a higher amount of added albumin. Orrego et al. [43] obtained aerated whey protein gels with a maximum hold-up of 70%, the lower value of this parameter was found for dispersion made at lower pH. Increasing gelatine concentration in gas-filled gels produced higher density and lower gas hold-up (in the range 72.9–64.3%) due to the higher viscosity of sol solution [44]. Karim and Wai [45] prepared the foams from starfruit puree with the addition of methylcellulose as a foaming agent. A higher concentration of foaming agent produced larger bubbles with a wide size distribution. However, at the highest concentration of the foaming agent, a slight decrease in bubble diameter was observed. Some of the air bubbles were more packed and could become elongated. Foamed apple juice with different concentration of albumin showed the decrease of bubble diameter with an increase of foaming agent concentration [10]. A similar trend was observed for an aerated gel with chokeberry juice concentrate. The addition of a higher amount of albumin caused the decrease of mean bubble diameter (Table 4). 

**Figure 1:**
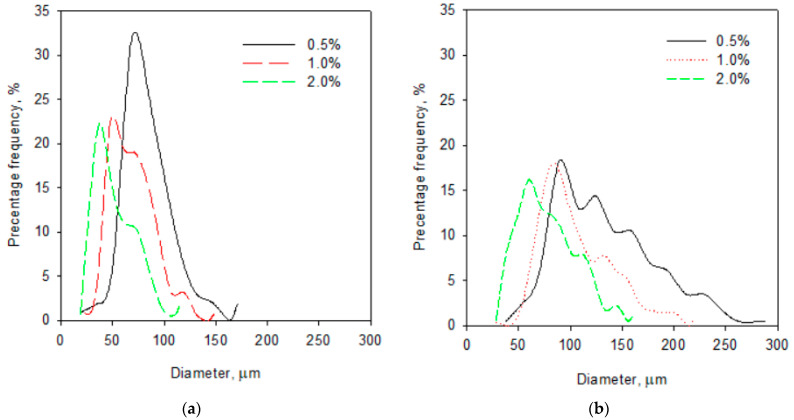
Diameter size distribution of bubbles in gels with 0.5, 1.0, 2.0% addition of albumin: (**a**) sample with 5% addition of concentrate; (**b**) sample with 20% addition of concentrate.

**Figure 2:**
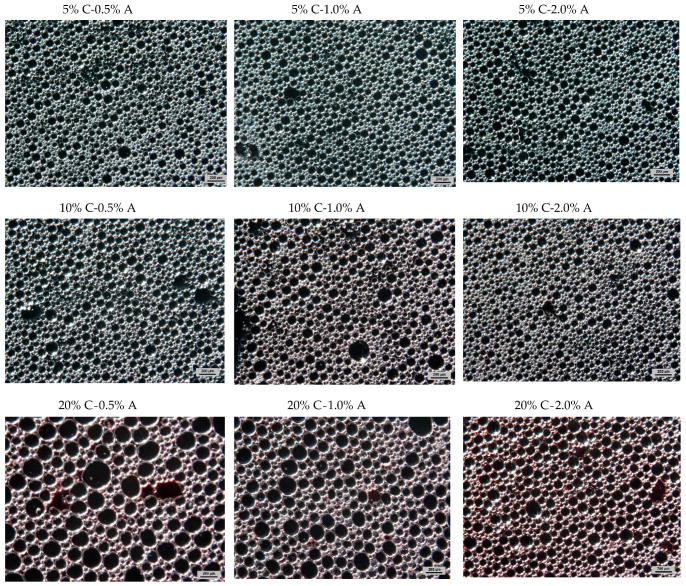
Images from optical microscopy of aerated gels with different compositions: C—chokeberry juice concentrate addition A—albumin addition; scale bar 200 µm.

Many factors such as type and concentration of foaming agent, pH, and viscosity of the continuous phase may affect the degree of aeration as well as dispersion and size of bubbles in aerated gel [43,44]. The present study shows that an increase of foaming agent concentration affected the creation of bubbles with smaller sizes and lower distribution of sizes (Figure 1 and Figure 2). The aerated gels with 20% addition of concentrate contained the bubbles with mean size in the range from 59.9 to 89.5 µm for albumin concentrations of 2 and 0.5%, respectively (Table 4). Figure 1b shows the bubbles size distribution in gels with 20% addition of CJC at different concentration of albumin. These gels contained bubbles ranging in diameter from 28 to 285 µm. The decrease of the addition of concentrate to 5% produced more homogenous samples (Figure 2) with smaller mean bubble diameter (range from 37.2 to 67.6 µm, Table 4) and narrow size distribution (range from 21 to 165 µm, Figure 1a). Some data for size distribution (for 10% addition of concentrate and 1.5% concentration of albumin) were not presented in Figure 1 to improve its clarity. The preliminary studies showed that incorporation of albumin to dispersion led to an increase of pH from 3.65 to 4.14 but the increase of addition of concentrate juice in sol caused a decrease of its pH to 3.57 (unpublished data). It can be assumed that the samples with higher pH (with the higher addition of albumin and lower addition of concentrate) enabled to obtain foamed gels with smaller bubbles which were more uniformly distributed within the product. Decreasing bubble size and avoiding polydispersity could minimise negative phenomena affecting the stability of aerated gels (e.g., vertical stratification) [44]. It may give a better appearance of the aerated gels.

**Figure 3:**
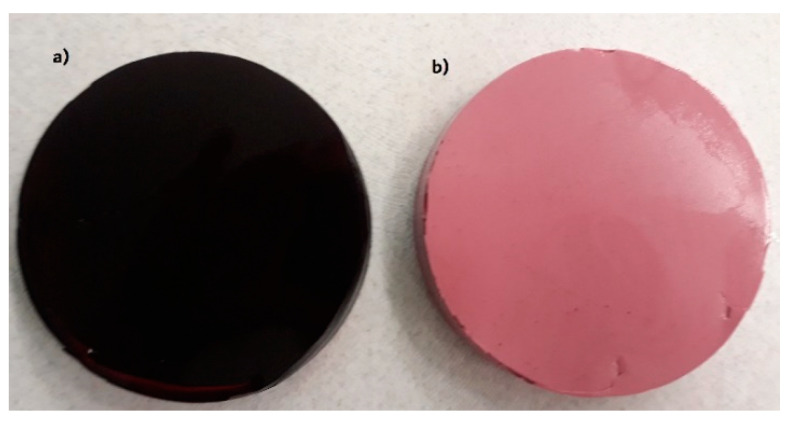
Images of non-foamed (**a**) and aerated with 1.5% of albumin (**b**) gels with addition of 20% chokeberry juice concentrate.

Table 5 shows the colour parameters obtained for aerated gels with a different addition of CJC. The lightness values L* of aerated samples were higher than non-foamed gels (Figure 3). The increase of albumin addition from 0.5 to 2.0% produced the brighter fabricated gel and it was observed for all foamed gel with different concentration of CJC. The incorporation of air bubbles affected the refraction of the light and the colour of foamed samples. The presence of bubbles also affected the colour attributes a* and b*, with increasing albumin concentration as the +a* (redness) and +b* values (yellowness) decreased. However, the increase of CJC in foamed gels (at the same albumin concentration) caused an increase of a* and b* values. The range of a* values (19.7–40.0) indicated the presence of red colourants in the aerated gels with CJC. Tolić et al. [25] observed that the a* parameter varied from 16.64 to 46.42 for some chokeberry juices, which is in the agreement with the present study. The b* values were positive, which indicated that the yellow colour was present in the foamed gels with CJC. Horszwald et al. [39] also reported the positive values of b* in the range from 1.27 to 4.50 for Aronia juice subjected to different drying techniques. The total colour difference ΔE between non-foamed gel and foamed material increased with increasing albumin concentration. The colour stability values ΔE showed that jelly and marshmallows with barberry fruit extract had higher stability than beet juice concentrate, which was used in the control samples as a natural red colourant [18]. 

**Figure 4:**
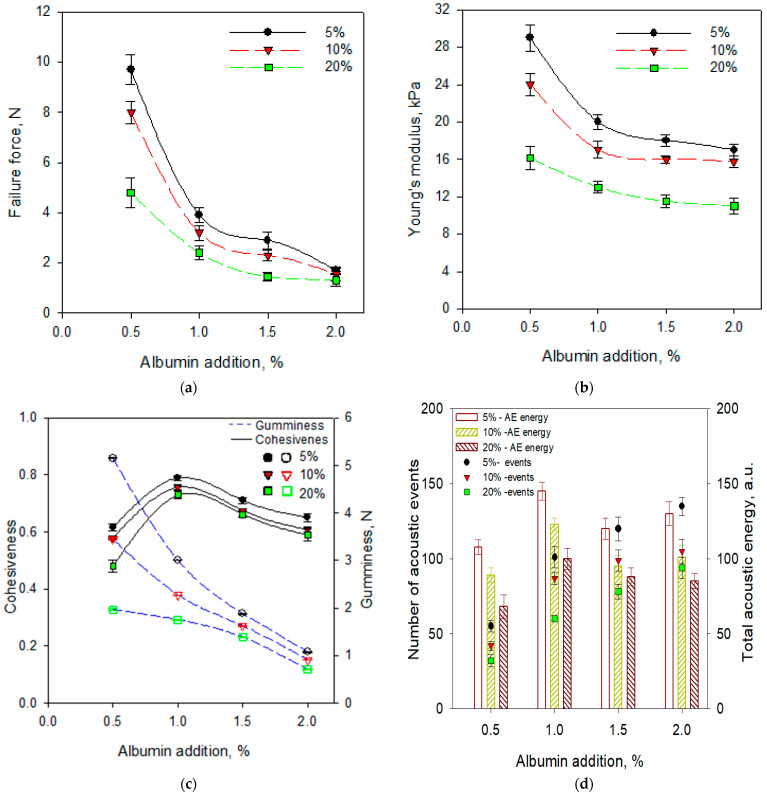
Selected mechanical and acoustic properties of gels with 5, 10, 20% addition of chokeberry juice concentrate and 0.5, 1.0, 1.5, 2% addition of albumin: (**a**) failure force; (**b**) Young’s modulus; (**c**) cohesiveness and gumminess; (**d**) total acoustic energy and number of acoustic events.

The mechanical properties of aerated gels at high deformation of samples showed that the failure force gradually decreased with increasing the addition of foaming agents (Figure 4a). The non-aerated samples were harder than foamed gels with CJC. Zúñiga and Aguilera [44] observed that aerated gelatine gels obtained by incorporation of different gas phases were weaker and less ductile than non-aerated gel due to the lower matrix content in the sample. The deformation of foamed gels at lower strain (in the range 30–37% for all concentrations of albumin and juice concentrate) showed that aerated gels had a more delicate and more brittle structure than non-foamed gels with juice concentrate. Young’s modulus also decreased with the addition of albumin as well as with an increase of the addition of CJC (Figure 4b). 

The gumminess and cohesiveness were determined for aerated gels (Figure 4c). The cohesiveness gradually increased with the increase of foaming agents to 1.0% of albumin addition but the higher concentration of albumin affected a decrease of the cohesiveness. The incorporation of a higher amount of strawberry pulp to kappa-carrageenan gels caused the decrease in cohesiveness, which was explained by interference of pulp particles in the formation of the gel matrix [46]. Gumminess significantly decreased with the increase of foaming agents. The changes of gumminess with an increase of foaming agent had a similar trend as observed for a force at fracture. Cohesiveness indicates coherence of a sample’s consistency. The aerated samples had higher values of cohesiveness than non-aerated gel, which may be related to different and more complex structures. Gumminess is related to the hardness and cohesiveness of the sample. Generally, the incorporation of bubbles in agar sol caused the decrease of the hardness of aerated gel, which led to reduce its gumminess.

The acoustic emission characteristic of samples depends on the composition of a structure of the deformed product. Some breaks and fractures generated during deformation can be a source of acoustic signals which contain information related to the structure and texture of food products [47]. The combination of mechanical and acoustic results can provide more knowledge about the texture of food products than any instrumental technique alone [48]. Total acoustic emission energy slightly increased with the addition of albumin to the concentration of 1% and then gradual decreased with a higher amount of foaming agent (Figure 4d). The lowest acoustic energy and number of acoustic events were observed during the deformation of samples with the highest addition of chokeberry juice concentrate. In addition, the number of acoustic events increased with the increasing addition of foaming agents. 

**Figure 5:**
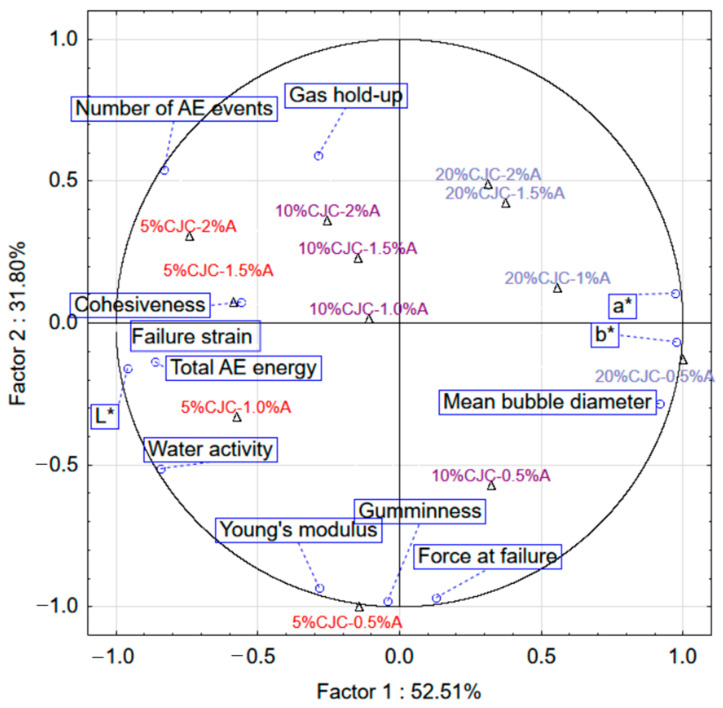
The first two components of PCA for selected properties of aerated gels with chokeberry concentrate juice (CJC) and with different albumin concentration (A).

The principal component analysis PCA was done to estimate the correlation between selected physical properties for aerated gels with CJC (Figure 5). The analysis indicated that 94.50% of the variability was explained by the three first components. The first two components together explained 84.50% of the variance. The mean bubble diameter and colour parameters a* and b* had a high loading factor (0.97–0.98) which corresponded to factor 1 of PCA. The TPA and mechanical parameters were characterised by factor loads that linked them with the negative part of factor 2 of PCA. Principal cluster analysis showed that the number of acoustic events and total acoustic energy were negatively correlated with the mean bubble diameter. It means that more acoustic energy and a higher number of acoustic events can be generated with the decrease of the size of bubbles. The reduction of bubble size also caused an increase in the number of bubbles (Figure 4). More small bubbles generated more crakes and acoustic events. Acoustic energy was also positively correlated with the failure strain and cohesiveness. The larger strain the longer distance to failure and that more acoustic energy can be generated. Figure 5 shows that the gels with CJC prepared with 0.5% addition of albumin had considerably different properties in comparison to gels with CJC prepared with a higher amount foaming agent. 

## 3. Conclusions

The addition of chokeberry juice concentrate changed the colour of agar gel with fructose, the attractive appearance of the aerated gel was also linked with the presence of fruit juice concentrate. It can be concluded that the obtained aerated gels were enriched with bioactive compounds derived from chokeberry juice concentrate. However, the addition of 20% of concentrate and foaming agent created samples with very low hardness, cohesiveness, gumminess as well as the structures of aerated samples were characterised by the larger bubble diameter and the wide distribution of its size. The more promising texture and structure properties were obtained for samples with aerated gels with 5 and 10% addition of chokeberry concentrate. Additionally, the patrial fructose replacement by chokeberry juice concentrate in gels can be positively assessed by consumers. The increase of albumin addition caused significant changes in the structure and texture of aerated samples. Generally, the incorporation of the higher concentration of foaming agents reduced the hardness of samples but their cohesiveness increased at a 1% addition of albumin. The higher the concentrations of egg albumin the more uniform and smaller the bubbles produced. The addition of 1% albumin to the gelled system led to obtaining the homogenous gelled structure with small bubbles and the incorporation of the higher amount of albumin to agar sol seems ineffective due to a significant decrease of hardness (a decrease of failure force), cohesiveness, and gumminess of gelled products. Further research is necessary to compare instrumental texture-structure properties with sensory analysis of obtained aerated products. 

## 4. Materials and Methods

The gels (non-foamed samples) were prepared by using the following ingredients: agar-agar powder (Hortimex Sp. z o.o., Konin, Poland), chokeberry juice concentrate (73.0 Brix) (Döhler, Natural Food and Beverage, Darmstadt, Germany) and fructose (Gadot Biochemical Industries Ltd., Haifa, Israel). The foamed gel also contained a required mass of albumin from chicken egg (Sigma-Aldrich Inc., St. Louis, MO, USA). The composition of non-foamed and foamed gels with CJC was presented in Table 6 and Table 7. All gels contained 55% sugars (sugars from juice concentrate and added fructose). The batch included the preparation of 400 mL dispersion of agar-sugar solution with or without CJC. The four batches were prepared for further experiments. The agar powder and fructose were dispersed in distilled water agitated and heated to 90 °C for 40 min. The mixture was cooled in the water bath until 50 °C. The chokeberry juice was added in the required amount to the agar solution and stirred. The obtained mixture was poured into plastic containers (80 mm diameter and 15 mm in height). The foamed gels were obtained by the addition of a required amount of egg albumin to dispersion and the mixture was whipped for 2 min at speed of 4000 rpm using a conventional kitchen type mixer (Severin, Sundern, Germany) with a whisk beater. The foamed dispersion was also poured on the same type of plastic containers. After 24 h of storage at room temperature, the samples were removed from the containers and diced into 15 mm cubes.

Water activity was measured using a Hygrolab C laboratory analyser (Rotronic, Bassersdorf, Switzerland) with a measurement accuracy of ±0.001. The measurement was repeated in triplicate.

The colour was determined in terms of CIE L*a*b* using a CR-111 200 colourimeter (Minolta, Osaka, Japan) with illuminant D65 and 10° observer. The measurement of colour was performed four times for four samples. The L* (lightness), a* (+a* redness/−a* greenness), b* (+b* yellowness/−b* blueness) values were analysed and the total colour change ΔE between non-foamed and foamed gels with CJC were calculated:

(1) $$\Delta E=\sqrt{{\left(L^{*}-L_{o}^{*}\right)}^{2}+{\left(a^{*}-a_{o}^{*}\right)}^{2}+{\left(b^{*}-b_{o}^{*}\right)}^{2}},$$

where L*_o_, a*_o_, b*_o_ are the colour attributes of non-foamed gel with CJC; L*, a*, b* are the colour attributes of foamed gels with CJC. The vales of ΔE were calculated for samples at the same concentration of CJC. 

The density of non-foamed gel as well gas hold-up was measured using the procedure described in the protocol for model fructose-gel [37]. The gas hold-up was calculated using the density of aerated gels ρ_f_ and non-foamed gel ρ_g_. The measurements were performed in triplicate.

(2) $$\mathrm{Gas}\;\mathrm{hold}-\mathrm{up}=\left(1-\frac{\rho_{f}}{\rho_{g}}\right).$$

The structure of foamed material was analysed using a stereoscope microscope (Nikon SMZ1500) equipped with a digital camera. The foamed samples were carefully spread on a glass slide and gently pressed down by cover-slip. The images (at least five pictures were recorded) were analysed using the auto-detect tool of the NIS-elements D3.1 software (Nikon Instruments Inc., Melville, NY, USA). The substantial number of bubbles (N = 600) were measured for each aerated sample to estimate bubble size distribution as well mean size of diameter according to the method applied by Raharitsifa et al. [10].

The compression tests were carried out using a TA-HD plus texture analyser (5-kg load cell) and flat-type probe (Stable Micro Systems, Godalming, Sy, UK). Samples (cubes with a side of 15 mm) were compressed at a constant speed of 1 mm∙s^−1^ and up to strain 70%. The force (N) and strain at fracture (%) were registered, also Young’s modulus (kPa) was calculated from the linear part of the compression curve. Twenty-five individual samples were subjected to a deformation test. The samples were also examined with Texture Profile Analysis (TPA) test using a TA-HD plus texture analyser (Stable Micro Systems, Godalming, Sy, UK). The test conditions involved two consecutive cycles of 30% compression with a time lag of 5 s between cycles. The deformation probe moved at a constant speed of 1 mm∙s^−1^. The TPA parameters cohesiveness and gumminess (N) were calculated from the deformation and force responses recorded using Exponent software analysis program (Stable Micro Systems, Godalming, Sy, UK). Each TPA measurement was replicated 25 times for all type of gels. 

The measurement of the acoustic emission (AE) was carried out during compressing the samples with a speed of 1 mm·min^−1^ and up to fracture. Acoustic emission was registered in the range 0.1–16 kHz using a piezoelectric accelerometer type 4381 (Brüel & Kjær, Naerum, Denmark). The total number of acoustic events and total acoustic energy (arbitrary unit- a.u.) were recorded and analysed according to the procedure described by Jakubczyk et al. [37]. Twenty-five replications of each type of sample were performed.

A one-way ANOVA test was used to analyse the significance of differences among samples at the 95% significance level. Significant pairwise differences were further tested using paired Tukey’s ‘Honest Significant Difference’ method. Principal component analysis (PCA) was carried out to describe multivariate relations between selected physical parameters of aerated gels with CJC. The data were standardised before PCA analysis and the results were presented as a biplot. The statistical analyses were performed with STATISTICA software v. 12.5. 

## Figures and Tables

**Table 1:** Selected physical properties of non-foamed gels with chokeberry juice concentrate.

Addition of Chokeberry Juice Concentrate, %	Water Activity	Density kg∙m^−3^	L*	a*	b*
0	0.678 ± 0.009 ^a,†^	1298 ± 60 ^a^	96.0 ± 1.1 ^a^	+2.0 ± 0.2 ^a^	+18.0 ± 0.4 ^a^
5	0.659 ± 0.010 ^a^	1322 ± 29 ^a^	24.0 ± 0.8 ^b^	+32.4 ± 0.9 ^b^	+1.1 ± 0.2 ^b^
10	0.634 ± 0.011 ^b^	1345 ± 24 ^a^	12.4 ± 0.9 ^c^	+35.0 ± 0.4 ^c^	+0.6 ± 0.2 ^b^
20	0.611 ± 0.013 ^b^	1358 ± 45 ^a^	4.1 ± 0.4 ^d^	+39.8 ± 0.3 ^d^	−0.4 ± 0.1 ^c^

^†^ the different letter (^a–d^) in the columns indicates the significant difference between the obtained values for samples, *p* ≤ 0.05.

**Table 2:** Mechanical descriptors and selected TPA parameters of non-foamed gels with chokeberry juice concentrate.

Addition of Chokeberry Juice Concentrate, %	Fracture Force, N	Fracture Strain, %	Young’s Modulus, kPa	Cohesiveness	Gumminess, N
0	48.3 ± 1.1 ^a,†^	59.6 ± 0.8 ^a^	63.2 ± 6.3 ^a^	0.74 ± 0.02 ^a^	11.7 ± 0.8 ^a^
5	37.4 ± 0.7 ^b^	53.7 ± 1.1 ^b^	52.6 ± 3.2 ^b^	0.58 ± 0.04 ^b^	4.9 ± 0.5 ^b^
10	21.2 ± 1.2 ^c^	47.2 ± 1.5 ^c^	39.0 ± 2.8 ^c^	0.61 ± 0.04 ^b^	3.7 ± 0.6 ^b^
20	7.8 ± 0.6 ^d^	39.2 ± 0.6 ^d^	18.8 ± 1.4 ^d^	0.44 ± 0.06 ^c^	1.9 ± 0.3 ^c^

^†^ the different letter (^a–d^) in the columns indicates the significant difference between the obtained values for samples, *p* ≤ 0.05.

**Table 3:** Acoustic properties of non-foamed gels with chokeberry juice concentrate.

Addition of Chokeberry Juice Concentrate, %	Total Acoustic Energy, a.u.	Number of Acoustic Events
0	259.4 ± 28.7 ^a,†^	101 ± 9 ^a^
5	194.4 ± 18.1 ^b^	55 ± 5 ^b^
10	148.1 ± 12.1 ^c^	32 ± 4 ^c^
20	3.2 ± 0.8 ^d^	13 ± 1 ^d^

^†^ the different letter (^a–d^) in the columns indicates the significant difference between the obtained values for samples, *p* ≤ 0.05.

**Table 4:** Selected physical properties of foamed gels with chokeberry juice concentrate.

Addition of Chokeberry Juice Concentrate, %	Addition of Albumin, %	Water Activity	Gas Hold-Up, %	Mean Bubble Diameter, µm
5	0.5	0.651 ± 0.009 ^a,†^	65.1 ± 1.2 ^ab^	67.6 ^a^
	1.0	0.650 ± 0.007 ^a^	61.2 ± 2.1 ^a^	47.2 ^b^
	1.5	0.648 ± 0.010 ^a^	68.7 ± 1.9 ^bc^	45.4 ^b^
	2	0.647 ± 0.011 ^a^	71.4 ± 2.0 ^c^	37.2 ^c^
10	0.5	0.632 ± 0.012 ^a^	67.1 ± 1.1 ^a^	77.8 ^a^
	1.0	0.631 ± 0.009 ^a^	68.2 ± 0.9 ^ab^	66.9 ^b^
	1.5	0.629 ± 0.010 ^a^	70.1 ± 1.3 ^bc^	58.0 ^c^
	2	0.627 ± 0.012 ^a^	71.2 ± 0.9 ^c^	54.4 ^c^
20	0.5	0.609 ± 0.013 ^a^	64.2 ± 1.1 ^a^	89.5 ^a^
	1.0	0.607 ± 0.014 ^a^	64.4 ± 0.7 ^a^	86.1 ^a^
	1.5	0.608 ± 0.009 ^a^	69.9 ± 0.8 ^b^	65.4 ^b^
	2	0.607 ± 0.011 ^a^	67.8 ± 1.1 ^b^	59.9 ^c^

^†^ the different letter (^a–c^) in the columns (for the same amount of added concentrate) indicates the significant difference between the obtained values for samples with different albumin concentration, *p* ≤ 0.05.

**Table 5:** Colour attributes of foamed gels with chokeberry juice concentrate.

Addition of Chokeberry Juice Concentrate, %	Addition of Albumin, %	L*	a*	b*	ΔE
5	0.5	61.6 ± 0.4 ^a,†^	+23.0 ± 0.1 ^a^	+1.5 ± 0.1 ^a^	38.1 ± 0.4 ^a^
	1.0	63.4 ± 0.3 ^b^	+22.0 ± 0.3 ^b^	+0.9 ± 0.1 ^b^	40.7 ± 0.6 ^b^
	1.5	65.3 ± 0.1 ^c^	+21.0 ± 0.4 ^b^	+0.7 ± 0.1 ^bc^	42.8 ± 0.3 ^c^
	2	69.1 ±0.5 ^d^	+19.7 ± 0.2 ^c^	+0.5 ± 0.1 ^c^	46.8 ± 0.5 ^d^
10	0.5	52.9 ± 0.4 ^a^	+29.1 ± 0.1 ^a^	+2.4 ± 0.1 ^a^	40.9 ± 0.2 ^a^
	1.0	54.6 ± 0.9 ^a^	+27.2 ± 0.2 ^b^	+1.8 ± 0.2 ^b^	42.9 ± 0.4 ^b^
	1.5	57.4 ±1.4 ^b^	+25.5 ± 0.7 ^c^	+1.1 ± 0.1 ^c^	46.0 ± 0.9 ^c^
	2	60.3 ± 1.2 ^c^	+22.4 ± 0.9 ^d^	+0.8 ± 0.1 ^c^	49.5 ± 1.0 ^d^
20	0.5	43.6 ±0.9 ^a^	+40.0 ± 0.3 ^a^	+3.8± 0.2 ^a^	39.7 ± 0.5 ^a^
	1.0	45.0± 1.6 ^ab^	+35.0 ± 0.7 ^b^	+3.0 ± 0.3 ^b^	41.3 ± 0.8 ^a^
	1.5	47.2 ± 1.3 ^bc^	+32.7 ± 0.8 ^c^	+2.6 ± 0.2 ^bc^	43.8 ± 0.5 ^b^
	2	49.1 ±0.4 ^c^	+31.0 ± 0.9 ^c^	+2.2 ± 0.1 ^c^	45.9 ± 0.6 ^c^

^†^ the different letters (^a–d^) in the columns (for the same amount of added concentrate) indicates the significant difference between the obtained values for samples with different albumin concentration, *p* ≤ 0.05.

**Table 6:** Composition of non-foamed gels with chokeberry juice concentrate.

Chokeberry Juice Concentrate, %	Agar Powder, %	Fructose, %	Water, %
0	1.5	55.0	43.5
5	1.5	51.4	39.8
10	1.5	47.7	42.2
20	1.5	40.4	38.1

**Table 7:** Composition of foamed gels with chokeberry juice concentrate.

Type of Foamed Gel	Chokeberry Juice Concentrate, %	Egg Albumin, %	Fructose, %	Agar Powder, %	Water, %
5% C-0.5% A	5	0.5	51.4	1.5	41.7
5% C-1.0% A	5	1.0	51.4	1.5	40.9
5% C-1.5% A	5	1.5	51.4	1.5	40.7
5% C-2.0% A	5	2.0	51.4	1.5	40.2
10% C-0.5% A	10	0.5	47.7	1.5	40.3
10% C-1.0% A	10	1.0	47.7	1.5	39.8
10% C-1.5% A	10	1.5	47.7	1.5	39.3
10% C-2.0% A	10	2.0	47.7	1.5	38.8
20% C-0.5% A	20	0.5	40.4	1.5	37.6
20% C-1.0% A	20	1.0	40.4	1.5	37.1
20% C-1.5% A	20	1.5	40.4	1.5	36.6
20% C-2.0% A	20	2.0	40.4	1.5	36.1

## Data Availability

The data generated or analysed during this study are available from the corresponding author on reasonable request.
